# The course of respiratory tract infections in pediatric hemato-oncology patients during and after the COVID-19 pandemic: single center retrospective cohort study

**DOI:** 10.3389/fped.2026.1703730

**Published:** 2026-02-04

**Authors:** Yeter Düzenli Kar, İmran Sağlık, Gökalp Rüstem Aksoy, Harun Ağca, Aytül Temuroğlu, Cem Uğur Mete, Melike Sezgin Evim, Büşra Safiye Beygo, Ahmet İbrahim Aydoğan, Çağlar Ödek, Solmaz Çelebi, Mustafa Hacımustafaoğlu, Güven Özkaya, Adalet Meral Güneş

**Affiliations:** 1 Division of Pediatric Hematology, Department of Pediatrics, Bursa Uludag University Faculty of Medicine, Bursa, Türkiye; 2 Department of Infectious Diseases and Clinical Microbiology, Bursa Uludag University Faculty of Medicine, Bursa, Türkiye; 3 Division of Pediatric Oncology, Department of Pediatrics, Bursa Uludag University Faculty of Medicine, Bursa, Türkiye; 4 Division of Pediatric Intensive Care Unit, Department of Pediatrics, Bursa Uludag University Faculty of Medicine, Bursa, Türkiye; 5 Division of Pediatric Infectious Diseases, Department of Pediatrics, Bursa Uludag University Faculty of Medicine, Bursa, Türkiye; 6 Department of Biostatistics, Bursa Uludag University Faculty of Medicine, Bursa, Türkiye

**Keywords:** after the COVID-19 pandemic, children, COVID-19 pandemic, hematologic-oncologic disease, respiratory tract infections

## Abstract

**Introduction:**

Respiratory tract infections (RTI) are a leading cause of hospitalization in children and remain a significant contributor to morbidity. Our study aimed to examine the epidemiology of RTI in children with hemato-oncologic disease during and after the coronavirus 2019 (COVID-19) pandemic.

**Methods:**

This retrospective study evaluated nasopharyngeal swab samples that were tested using multiplex PCR from 185 children hospitalized with respiratory symptoms between January 2020 and March 2025.

**Results:**

A total of 313 RTI agents were identified in 185 children with hemato-oncologic disorders across 271 infectious episodes. The median age was 6 years. The infection rates for upper respiratory tract infections (URTI) and lower respiratory tract infections (LRTI) were 45% and 55%, respectively. A statistically significant difference was found between patients with URTI and LRTI in terms of CRP, tachypnea, dyspnea, duration of fever, duration of hospital stay due to infection, need for intensive care unit, and oxygen requirements (*p* < 0.05). The most common pathogens causing RTI were *rhinovirus/enterovirus* and *influenza A/B*, and their frequencies increased significantly in the post-pandemic period. Co-infections were significantly more common in the LRTI group during the post-pandemic period. Nine out of 185 children (5%) died.

**Conclusion:**

Children with hematologic-oncologic disease are at risk for RTI. The most common agent was found to be *rhinovirus/enterovirus*. RTI outbreaks were observed especially as a result of the reductions in non-pharmacological measures implemented during the pandemic. In our study, the largest RTI outbreak was seen between November 2023 and May 2024 following the end of the COVID-19 pandemic.

## Introduction

In healthy children, respiratory viruses usually have a self-limiting, mild clinical course in the form of upper respiratory tract infection (URTI). However, in immunocompromised children receiving myelosuppressive therapy and/or hematopoietic stem cell transplantation (HSCT), they can progress to lower respiratory tract infection (LRTI) and cause widespread and severe disease (1). Therefore, despite advances made in childhood cancer treatment outcomes in the last decade, respiratory tract infections (RTI) and their complications can negatively affect treatment outcomes (2).

During the coronavirus disease 2019 (COVID-19) outbreak, some universal practices were implemented to protect public health around the world in order to prevent COVID-19 transmission. These included public education about universal mask use in public areas, the obligation to pay attention to hand hygiene, and infection control measures such as quarantine, social distancing (closure-partial closure), and occupational health policies for sick healthcare workers (being considered on leave during the illness) (3, 4). Therefore, there was a significant decrease in the incidence of respiratory viruses other than COVID-19 worldwide with the pandemic (5). Since May 5, 2023, the date when the World Health Organization (WHO) officially announced the end of the COVID-19 pandemic, an increase in non-COVID-19 viral respiratory infections has been reported (6). In our country, the most frequently detected respiratory viral agents in pediatric cancer patients in the pre-pandemic period were reported to be *rhinovirus/enterovirus, parainfluenza (PIV), respiratory syncytial virus (RSV),* and *influenza* (7, 8). There are limited data evaluating the distribution of viral agents in children during three different time periods; the pandemic, relaxation of pandemic measures and after the COVID-19 outbreak in various disease settings.

The aim of the study is to evaluate the changes in viral RTI agents after COVID-19 compared to the lockdown period and to investigate the associated clinical findings, complications and mortality data for our pediatric hematology and oncology patients.

## Material and method

### Study design and data collection

In this study, nasopharyngeal swab samples were taken from children with signs and symptoms of acute RTI hospitalized at Bursa Uludag University Sabahattin Gazioğlu Pediatric Hematology and Oncology Hospital between 01.01.2020 and 01.03.2025. The study included patients aged <23 years old, who either had cancer treatment within the last six months, or received HSCT with ongoing immunosuppressive therapy. Children with benign immunosuppressive hematological disorders were also included in the study. Patients considered immunosuppressive with benign hematological diseases were those using long-term high-dose corticosteroids and/or immunosuppressive drugs such as cyclosporine due to immune cytopenia (anemia, thrombocytopenia), patients with congenital hemolytic anemia (thalassemia, hereditary spherocytosis) who had splenectomy and/or receive regular blood transfusions, and patients with common variable immune deficiency, combined immune deficiency and congenital neutropenia.

RTI was defined as episodes with fever ≥38°C at least once in the previous 72 h, and also one of the symptoms of cough, sore throat, nasal congestion, runny nose, or respiratory distress. Age, gender, diagnosis of hemato-oncological disease, date of onset of symptoms and hospitalization, duration of hospital stay, duration of fever, presence of tachypnea/dyspnea, need for intensive care, duration of hospitalization due to infection, concomitant blood-urine-catheter-stool culture positivity, other concomitant infection focus, white blood cell count (WBC), neutrophil, lymphocyte, monocyte and platelet counts, C-reactive protein (CRP), and final status (alive/died) of the patients were evaluated based on their electronic hospital records.

Respiratory tract infectious episodes were divided into two groups; URTI and LRTI. URTI was defined as the presence of at least one of the following symptoms: cough, fever, sore throat, runny nose, or nasal congestion, without abnormal chest examination findings. LRTI was described as the presence of findings indicating lower respiratory tract involvement on chest examination (tachypnea, oxygen requirements, rales, rhonchi, bronchial breathing sounds, etc.), and/or signs of new pulmonary infiltrates observed on chest radiography and/or computed tomography (CT).

We divided the COVID-19 pandemic and afterwards into three time periods;

- Lockdown period: Between January 2020 and May 2021 including the beginning of the pandemic and lockdowns and distance learning at schools in Türkiye.
- Relaxation Period: Between May 2021 and May 2023 when rules were loosened after the lockdowns.
- After the end of COVID-19: Between 5 May 2023 and March 2025.

### Supportive care

All patients were placed in protective isolation in single rooms. All immunosuppressed patients received cotrimoxazole administered for consecutive 2 days a week as prophylaxis for *Pneumocystis carinii* pneumonia from the start of immunosuppressive treatment until three months after the discontinuation of immunosuppression. HSCT recipients received acyclovir prophylaxis against CMV infection until the discontinuation of immunosuppression. Patients with neutrophil counts <100/mm^3^ for more than seven days were given antifungal prophylaxis until the neutrophil count was >500/mm^3^ (9). Intravenous immunoglobulin was administered if the patient immunoglobulin G levels were <500 mg/dL and immunoglobulin G levels were monitored every 3 weeks (10). The febrile neutropenic phase was treated with broad-spectrum antibiotics and this treatment was modified subsequently according to the results of blood or tissue cultures (9, 10).

### Sample collection and screening

This study included patients who were hospitalized at Bursa Uludağ University Children's Hematology and Oncology Hospital, which is the largest referral center in the south Marmara region with a population of nearly six million. Nasopharyngeal swab specimens were transported to the department of microbiology laboratory in a transport medium (UTM-RT, Copan, Italy) and were tested using the fully automated multiplex polymerase chain reaction (PCR)-based BioFire FLOQ Swabs (Copan, Italy). The respiratory pathogens were identified using the QIAStat Dx Respiratory SARS-CoV-2 (COVID-19) Panel (Qiagen, Germany) on the QIAStat Dx system (Qiagen, Germany) from 2020 to 2021, followed by the BioFire FilmArray Respiratory Panel (bioMérieux, Marcy-l'Étoile, France) on the FilmArray 2.0 System (bioMérieux) from 2021 to February 2025. These panels are multiplex PCR-based syndromic testing platforms capable of detecting respiratory pathogens simultaneously. Both panels included *influenza A, influenza B,* and *influenza A H1N1 2009, rhinovirus/enterovirus, PIV 1,2,3,4, coronaviruses (CoVs) NL 63, 229E, 0C43, HKU1 and COVID-19, human metapneumovirus (MPV) A/B, RSV A/B, adenovirus, Mycoplasma pneumoniae* and *Bordetella pertussis*. However, only *bocavirus* and *Legionella pneumophila* can be detected by the QIAStat Dx respiratory panel, and only *Bordetella parapertussis* and *Chlamydophila pneumoniae* can be detected by the BioFire FilmArray panel. Detection of more than one agent at the same time in the PCR panel in the same person was considered co-infection. If the same patient was re-infected with the same virus one month after complete clinical recovery, this event was accepted as a second episode. If the patient continued to have RTI symptoms without any clinical improvement, a repeat PCR panel was obtained in the third week of the infection to assess whether the viral agent persisted or whether a new agent had been added to the picture. If the same agent was still detected after one month, persistent infection was considered in the patient.

### Ethics committee

This retrospective study was conducted using anonymized patient data from electronic medical records. Institutional Review Board of Bursa Uludag University Health Research Ethic Committee on 19.03.2025 with decision number 2025/558-6/22. Written informed consent was obtained from the parents of patients who participated in this study.

### Statistics

Mean, standard deviation, median, minimum-maximum values, frequency, and percentage were used for descriptive statistics. The distribution of variables was checked with the Kolmogorov–Smirnov test. The independent samples t-test and Mann–Whitney U test were used to compare quantitative data. The chi-square test was used for the comparison of qualitative data. A forward stepwise multivariate binary logistic regression model was constructed at the patients' episodes level (*n* = 271 episodes), incorporating age, gender, diagnosis, HSCT status, neutropenia, pathogen type, and pandemic period to estimate adjusted associations with outcomes. Moreover, the multivariable binary logistic regression analysis was constructed at the patient level (*n* = 185 patients), incorporating age, gender, diagnosis, HSCT status, with only the first recorded infectious episode per patient included to avoid within-subject correlation. A backward stepwise elimination procedure was applied to identify independent predictors of the outcome. Gender and diagnosis categories were initially entered into the model, and variables were sequentially removed based on likelihood-ratio criteria to identify independent predictors of the outcome. All tests adopted a value of *p* ≤ 0.05 for statistical significance. Analyses were conducted using SPSS (version 22.0).

## Results

In this study, 271 RTI episodes in 185 children with hemato-oncologic disorders were analyzed. The median age of the patients was 6 years (range: 4 months–23 years), with a female-to-male ratio of 85:100. Episodes were divided into four groups according to patient diagnosis (Table 1).

**Table 1:** Characteristics of patients during URTI and LRTI infections.

Variables (All episodes)		URTI (*n* = 122)	LRTI (*n* = 149)	*p*
Age, year		5 (0.3-21)	7 (0.3-23)	0.062
Gender	Female	61 (50%)	64 (43%)	0.247
	Male	61 (50%)	85 (57%)	
Diagnosis at episodes	Leukemia/MDS/JMML	52 (42.6%)	73 (49%)	0.051
	Solid tumor	43 (35.2%)	44 (29.5%)	
	HSCT	3 (2.5%)	13 (8.7%)	
	Benign hematologic disorders	24 (19.7%)	19 (12.8%)	
Laboratory				
White blood cell count (/mm3)		3,800 ± 4,107	5,121 ± 13,619	0.303
White blood cell count category	Leukopenia (<4,000)	90 (32.2%)	104 (38.4%)	0.719
	Normal (4,000–10,000)	24 (8.9%)	32 (11.8%)	
	Leukocytosis (>10,000)	8 (3%)	13 (4.8%)	
Absolute neutrophil count (/mm3)		1,834 ± 2,580	2,530 ± 6,123	0.242
Neutropenia status	Neutropenic	76 (28%)	95 (35.1%)	0.804
	Non-neutropenic	46 (17%)	54 (19.9%)	
Absolute lymphocyte count (/mm3)		1,329 ± 1,575	1,850 ± 7,528	0.453
Lymphopenia status	Lymphopenic	90 (33.2%)	118 (43.5%)	0.293
	Non-lymphopenic	32 (11.8%)	31 (11.4%)	
Monocyte count (/mm3)		470 ± 719	539 ± 789	0.460
HGB (g/dL)		9.5 ± 1,9	9.1 ± 2.05	0.114
Thrombocyte count (/mm3)		173.525 ± 145.647	164.315 ± 164.053	0.630
Eosinophil count (/mm3)		71 ± 142	52 ± 126	0.262
CRP(mg/L)		32 ± 38	56 ± 78	0.001
Fungal co-infection		1 (0.8%)	4 (2.7%)	0.383
Bacteriemia		0	9 (6%)	0.005
Clinical findings				
Cough		48 (39.7%)	75 (50.3%)	0.080
Fever		99 (81%)	129 (86.6%)	0.224
Tachypnea, dyspnea		2 (1.6%)	30 (20.1%)	<0.001
Fever duration		2 (1–11)	2 (1–21)	0.015
Infection-related length of stay		7 (1–28)	8 (1–45)	0.001
Intensive care requirement		–	13 (8.7%)	–
Oxygen requirements	Nasal Mask	–	11 (7.4%)	–
	High-flow nasal oxygenation	–	2 (1.3%)	
	Invasive mechanical ventilation	–	11 (7.4%)	
All infectious episodes	Lockdown period	10 (43.4%)	13 (56.6%)	0.984
	Relaxation period	41 (45.5%)	49 (54.5)	
	After the end of COVID19 outbreak	71 (45%)	87 (55%)	
Infectious episodes	*Rhinovirus/enterovirus*	57 (46.7%)	54 (36.2%)	0.018*
	*Influenza Type A/B*	17 (13.9%)	12 (8.1%)	
	*COVID-19*	8 (6.6%)	19 (12.8%)	
	*Respiratory syncytial virus*	10 (8.2%)	16 (10.7%)	
	*Parainfluenza*	11 (9.0%)	5 (3.4%)	
	*Adenovirus*	5 (4.1%)	4 (2.7%)	
	*Coronavirus (non-COVID 19)*	3 (2.5%)	6 (4.0%)	
	*Metapneumovirus*	1 (0.8%)	1 (0.7%)	
	*Bocavirus*	0 (0%)	1 (0.7%)	
	*Bordetella Pertussis*	1 (0.8%)	1 (0.7%)	
	*Mycoplasma Pneumonia*	0 (0%)	1 (0.7%)	
	Co-infection	9 (7.4%)	29 (19.5%)	
Co-infection episodes^a^	Lockdown period	2 (1.6%)	3 (2.0%)	1.000
	Relaxation period	0 (0%)	8 (5.4%)	0.009
	After the end of COVID19 outbreak	7 (5.7%)	18 (12.1%)	0.073

Descriptive statistics given as mean, standard deviation, median (minimum-maximum), frequency, and percentage.

COVID-19: coronavirus disease 2019, MDS: Myelodysplastic syndrome, JMM: Juvenile myelomonocytic leukemia, HGB: Hemoglobin, CRP: C reactive protein.

a Co-infection; The most common co-infection agents were Rhino/enterovirus + COVID-19 (*n* = 7), Rhinovirus/enterovirus + parainfluenza (*n* = 3), Respiratory syncytial virus + COVID-19 (*n* = 3).

* Only both co-infection and Parainfluenza infectious episodes were statistically significant.

Group 1 (*n* = 77 patients): RTI episodes in children with hematologic malignancies *n* = 125 episodes (46%); acute lymphoblastic leukemia (ALL) 36.9% (*n* = 100), acute myeloblastic leukemia (AML) 4.4% (*n* = 12), relapsed ALL 3.3% (*n* = 9), myelodysplastic syndrome (MDS) (*n* = 2), relapsed AML (*n* = 1), juvenile myelomonocytic leukemia (JMML) (*n* = 1).

Group 2 (*n* = 67 patients): RTI episodes in children with solid tumors *n* = 87 (32.1%) (*n* = 67 patients).

Group 3 (*n* = 12 patients): RTI episodes in those receiving immunosuppressive therapy following HSCT, *n* = 16 (5.9%).

Group 4 (*n* = 29 patients): Other RTI episodes; *n* = 43 (15.9%) in those with benign hematologic diseases (thalassemia, hereditary spherocytosis, congenital neutropenia, immunodeficiency, immune cytopenia).

Of the 271 episodes, URTI and LRTI were detected in 45% (*n* = 122/271) and 55% (*n* = 149/271), respectively. There was no statistically significant difference between the URTI and LRTI groups in terms of WBC, neutrophil, lymphocyte, monocyte, hemoglobin, and platelet counts (*p* > 0.05). The only difference was found for CRP, which was significantly higher in the LRTI group (*p* = 0.02) (Table 1). The clinical findings, in terms of tachypnea, dyspnea, duration of fever, duration of hospital stay due to infection, need for intensive care unit, and oxygen requirements, were also significantly different between the two groups (*p* < 0.05) (Table 1). Fungal co-infection was present in five of the episodes and bacteremia co-infection was present in nine. Of the 271 episodes, 40% (*n* = 111/271) were *rhinovirus/enterovirus* infection which was followed by *influenza* 10.7% (*n* = 29/271), *COVID-19* 10% (*n* = 27/271), and co-infections 14% (*n* = 38/271). The frequency of co-infections was significantly higher in those with LRTI compared to URTI (*p* = 0.018) (Table 1). The frequency of co-infections increased gradually and reached its highest value in the post-pandemic period (lockdown 5/38, relaxation 8/38, after the outbreak 25/38) (Table 1).

When co-infection episodes are evaluated, co-infections were rare during the lockdown period. In the relaxation period, LRTI cases had a significant increase (8/149, 5.4%; *p* = 0.009). After the end of the COVID-19 outbreak, co-infection rates rose in both groups (URTI 7/122, 5.7%; LRTI 18/149, 12.1%), with a trend toward higher rates in LRTI that did not reach statistical significance (*p* = 0.073) (Table 1).

*PIV* was more frequent in the URTI group (*p* < 0.05). No statistically significant difference was found for other agents in terms of causing upper or lower RTI (Table 1).

In the adjusted multivariate logistic regression model that accounted for age, gender, diagnostic category, HSCT status, neutropenia, pathogen type, co-infection status, and pandemic period, HSCT status emerged as the only independent predictor of developing LRTI (*n* = 185 patients = 271 episodes). HSCT recipients episodes (*n* = 16/271 episodes) and HSCT recipients (*n* = 12/185 patients) demonstrated significantly higher odds of developing LRTI compared with leukemia/MDS/JMML patients (episodes: OR = 4.05, 95% CI 1.06–15.44, *p* = 0.040; patients: OR = 11.29, 95% CI = 1.32–96.70, *p* = 0.027). Although co-infections showed a trend toward increased risk for developing LRTI in episodes (*n* = 38/271) (OR = 4.60, 95% CI 0.97–21.92, *p* = 0.055), this did not reach statistical significance, and no other diagnostic or pathogen categories were independently associated with LRTI after adjustment.

A total of 313 infectious agents were identified in 185 children across 271 infectious episodes. The detected pathogens are given in Table 2. We found that the infectious episodes in total increased significantly after the end of pandemic (*p* < 0.001) (Table 2). The only significant increase among the all pathogens was observed for *influenza A/B* (lockdown period; *n* = 5, 17.9%, after the outbreak; *n* = 30, 16%) and rhinoviruses (lockdown period; *n* = 12, 43%, relaxation period; *n* = 52, 53.6%, after the outbreak; *n* = 70, 37.2%, *p* < 0.001). Although no statistically significant difference was detected for COVID-19, most cases were seen in the post-outbreak period (Table 2).

**Table 2:** Viral infection agents.

All infectious agents	Lockdown period *n* (%)	Relaxation period *n* (%)	After the end of the COVİD19 outbreak *n* (%)	Total *n* (%)	*P*
*Rhinovirus/ Enterovirus* *	12 (43.0)	52 (53.6)	70 (37.2)	134 (42.8)	<0.001*
*COVID-19*	2 (7.1)	17 (17.5)	22 (11.7)	41 (13.1)	
*Influenza type A/B* *	5 (17.9)	2 (2.1)	30 (16.0)	37 (11.8)	
*Respiratory syncytial virus*	2 (7.1)	17 (17.5)	17 (9.0)	36 (11.5)	
*Parainfluenza*	2 (7.1)	3 (3.1)	18 (9.6)	23 (7.3)	
*Adenovirus*	2 (7.1)	2 (2.1)	10 (5.3)	14 (4.5)	
*Coronavirus (non-COVID-19)*	2 (7.1)	3 (3.1)	8 (4.3)	13 (4.2)	
*Mycoplasma pneumonia*	0 (0.0)	0 (0.0)	6 (3.2)	6 (1.9)	
*Bordetella pertussis*	0 (0.0)	0 (0.0)	4 (2.1)	4 (1.3)	
*Metapneumovirus*	0 (0.0)	0 (0.0)	3 (1.6)	3 (1.0)	
*Bocavirus*	1 (3.6)	1 (1.0)	–***	2 (0.6)	
Total	28 (8.9)	97 (31)	188 (60.1)	313 (100)	<0.001**

* A significant difference Rhinovirus/ Enterovirus and Influenza type A/B.

** A significant difference all periods.

*** PCR kit did not contain Bocavirus and not studied.

Using two different PCR kits can introduce inconsistencies in pathogen detection. *Bocavirus* was detected in one patient during the lockdown period and in one patient during the relaxation period. We were aware that the PCR kit did not contain *Bocavirus* at the time it was changed. However, we did not conduct any additional studies because supportive care is recommended since there is no specific treatment.

Twelve patients (6.5%) out of 185 children with LRTI required intensive care unit (ICU) admission, of whom only one child was admitted to the ICU twice. Therefore, 13 episodes (5%; *n* = 13/271) were monitored in the ICU. In this cohort, 9 out of 185 children (5%) died of LRTI. They all required intensive care prior to death. Only 3 of these patients died secondary to RTI. None of these patients had progressive disease or other lethal conditions. Two patients died due to rhino/enterovirus pneumonia and one patient died due to COVID-19 pneumonia. Three of the other six patients had progressive disease accompanying the viral infection, one had cytomegalovirus (CMV) infection, one had lung graft-vs.-host disease (GvHD), and one had intracranial hemorrhage (Table 3). Although episodes in HSCT patients comprised 5.9% (*n* = 16/271) of all episodes, the majority had LRTI (*n* = 13/16), and approximately 19% (*n* = 3) required intensive care. Two of these patients died (Tables 1, 3).

**Table 3:** Clinical and laboratory findings of children in ICU.

No	Age (year)	Infection date	Gender	Agents	Bacterial or fungal or viral coinfections	Diagnosis	Neutrophil count (/mm3)	Lymphocyte count (/mm3)	Monocyte count (/mm3)	C-Reactive Protein (mg/L)	Last stiuation	Causes of death
1	8	10.02.2020	Male	*Rhinovirus/ Enterovirus*		ALL + HSCT + GvHD	1.443	565	446	2	Ex	LRTI + Lung GvHD
2	2	07.08.2020	Female	*Adenovirus* *+* *Parainfluenza Virus* *+* *Rhinovirus/Enterovirus*	CMV viremia	Combined Immunodeficiency	1.930	144	229	144	Ex	LRTI + CMV viremia
3	6	18.08.2020	Male	*Rhinovirus/Enterovirus*	–	Hodgkin lymphoma	1.620	1.210	1.706	54	Ex	LRTI
4	13	08.11.2021	Male	*Covıd-19*	–	AML	0	190	16	255	Ex	LRTI
5	3	11.04.2022	Female	*Rhinovirus/ Enterovirus*	–	MDS	370	2.380	2.480	21	Ex	LRTI + intracranial hemorrhage
6	4	16.12.2022	Male	*Rhinovirus/ Enterovirus* *+* *Covıd-19*	–	ALL + HSCT + GvHD	11.830	880	474	57	Alive	–
7	13	16.03.2023	Male	*Rhinovirus/ Enterovirus*	–	AML following MDS	9.100	1.650	2.780	18	Alive	–
7	13,5	20.09.2023	Male	*Rhinovirus/ Enterovirus*	–	AML following MDS	540	1.100	178	51	Ex	LRTI
8	12	29.01.2024	Female	*Covıd-19*	–	Ewing Sarcoma	6.200	280	669	157	Ex	LRTI + progressive disease
9	16	12.11.2024	Female	*Rhinovirus/ Enterovirus, Mycoplasma Pneumonia*	–	ALL	3.520	300	362	278	Alive	–
10	4	28.11.2024	Male	*Rhinovirus/ Enterovirus*	–	Combined Immunodeficiency + HSCT + GvHD	14.900	330	826	53	Alive	–
11	13	06.12.2024	Female	*Respiratory Syncytial Virüs* *+* *Rhinovirus/ Enterovirus,*	Stenotrophomonas maltophilia + Mucorales spp. in the nasal septum	Anaplastic ependimoma	720	180	91	463	Ex	LRTI + progressive disease
12	14	13.01.2025	Female	*Rhinovirus/ Enterovirus,*		Pineoblastoma	8.340	710	249	381	Ex	LRTI + progressive disease

ICU, intensive care unit; GvHD, graft vs. host disease; LRTI, lower respiratory tract infections; MDS, myelodisplastic syndrome; AML, acute myeloid leukemia; ALL, acute lymphoblastic leukemia; CMV, cytomegalovirus.

## Discussion

Uludağ University Hospital, located in Bursa, Türkiye's fourth-most populous city, is the only reference center in the southern Marmara region that offers solid organ and bone marrow transplants and treatment for hemato-oncological disorders. With a population of approximately six million and proximity to Istanbul, the southern Marmara region was significantly impacted by the pandemic (11).

Most of the RTI data for the *COVID-19* pandemic comes from the period of the outbreak. There is scarce data available afterwards (12–17). Epidemiological data about RTI in children with cancer from Türkiye were examined during the pandemic (13). This study is particularly important because it examines the distribution and pathogenic properties of RTI in detail and compares data during and after the pandemic covering the entire southern Marmara region.

**Figure 1:**
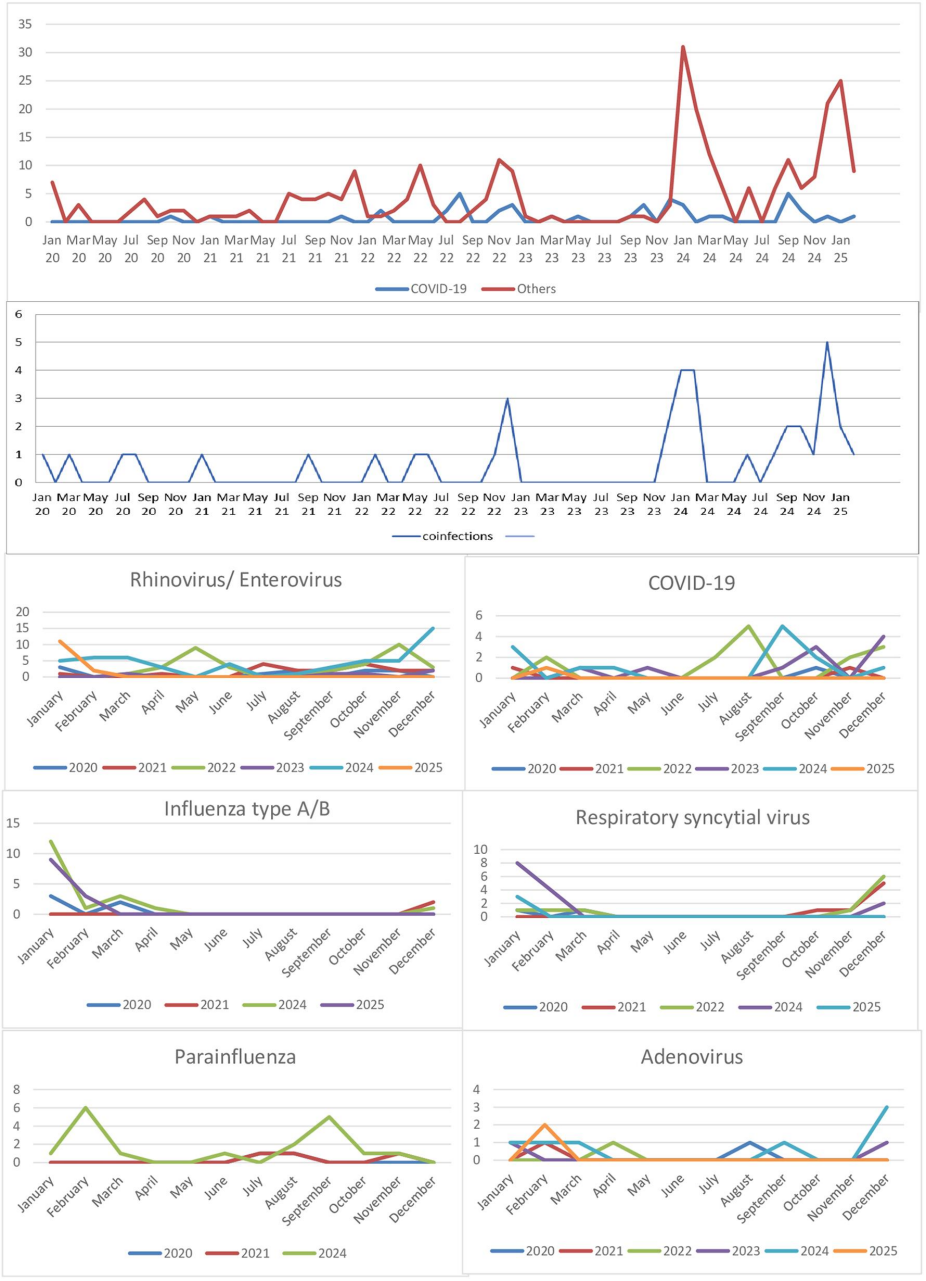
Seasonal distribution of RTI.

We found that the frequency of all viral agents, including *COVID-19*, was low during the pandemic between March 2020 and May 1, 2021, when the pandemic rules were strictly implemented in Türkiye (Figure 1; Table 2). During this period, only two of our patients were hospitalized due to *COVID-19*. We believe that the reason fewer *COVID-19* and other RTI agents were detected is the effective implementation of non-pharmacological measures. Current treatment options for RTI largely involve supportive care. Protection from respiratory viruses is crucial. Therefore, it is vital for patients and their caregivers/close contacts to adhere to preventive measures, such as wearing masks, maintaining social distancing, and practicing hand hygiene. Annual influenza vaccination is also recommended for all immunocompromised patients aged six months and older, as well as those in close contact with patients. Early diagnosis, careful monitoring, and appropriate treatment for RTI are crucial to prevent potential serious complications (10, 13). The influenza vaccination status of our patients and their close contacts was unknown.

In addition, patients without severe and persistent fever and/or LRTI findings and/or severe neutropenia were closely monitored by telemedicine without hospitalization. If possible, chemotherapy was usually administered in the outpatient unit with outpatient follow-up. All patients requiring hospitalization were subjected to a *COVID-19* test along with their companions before hospitalization, and they were admitted if the test result was negative. Other studies also reported similar results in that both *COVID-19* and other RTI decreased significantly in the first months of the *COVID-19* outbreak due to strictly applied pandemic measures (15, 18–20). The decrease in all RTI agents during the pandemic led to a decrease in immunity in the entire society, creating an “immunity gap”. For this reason, the frequency of some viral agents has significantly increased and more severe clinical courses occurred with the relaxation of non-pharmacological measures (16–18, 21). Our findings showed a gradual increase in all infection agents (Table 2). *Rhinovirus/enterovirus* was the most frequent agent detected in all time periods. Specifically, significant increases in the frequency of *rhinovirus/enterovirus* and *influenza A/B* were observed following the relaxation of pandemic regulations (Table 2). In a study examining 319 nasopharyngeal swab samples from 0 to 92 year olds in our hospital during the period, *rhino/enterovirus* was the most commonly identified causative agent during the pandemic (11).

While viruses cause URTI in the general population, they also cause LRTI in hemato-oncology patients (22). In our cohort, the frequency of URTI and LRTI was similar in all periods (*p* = 0.984) (Table 1). However, co-infections were significantly more common in the LRTI group, and parainfluenza frequently caused URTI (Table 1). After the end of the COVID-19 outbreak, co-infection rates increased in both upper and lower groups, with a trend toward higher rates in LRTI that did not reach statistical significance (Table 1). The literature reports that the frequency of co-infections increased after the pandemic, and that there was no relationship between co-infections and LRTI (13, 23). CRP and clinical findings supporting the diagnosis of LRTI, such as tachypnea, dyspnea, duration of fever, and duration of hospital stay due to infection, were also found to be significantly higher (*p* < 0.05) (Table 1). The need for intensive care and oxygen requirements were not present in URTI cases (Table 1).

Since HSCT recipients among hemato-oncology patients were more immunosuppressive, in our study, in line with the literature, HSCT recipients were found to be more susceptible to LRTI compared to other patient groups (*n* = 12/185 patients, *p* = 0.027) (13, 24).

Thirteen episodes (5%; *n* = 13/271) were monitored in the ICU. In our study, 3% of all episodes (*n* = 9/271) resulted in death, which corresponds to 5% (*n* = 9/185) of all children (Table 3). It is difficult to attribute these deaths to RTI because of other comorbidities. Six of the 9 children who died had underlying conditions and they all had LRTI. Kaçar et al. (13) reported that 28.7% of all episodes were LRTI in Türkiye. However, the frequency of LRTI in our cohort was high (55%) and it is also noteworthy that all those who died had LRTI. The LRTI rate may have been higher because our study only evaluated inpatients. The death rate in the study by Kaçar et al. (13) was slightly higher than in our study, but it included only the COVID-19 pandemic period. In this cohort, two patients out of 41 (4.8%; *n* = 2/41) died from COVID-19 infection (Table 3). A study in Türkiye by Rejin Kebudi et al. (24) reported a death rate of 2% (*n* = 1/51) related to COVID-19 in 51 children with cancer and stem cell transplant recipients. COVID-19 infection generally progressed with mild clinical symptoms during the pandemic. It was not reported as the most lethal RTI agent during or after the pandemic (4, 18, 25–30). In a study conducted in China about 285 pediatric patients with hematological malignancies, 89.1% of the patients had mild infection course while 1.8% had severe course, and 0.7% had critical course and died (30). In meta-analysis and cohort studies conducted in children with hemato-oncological diseases, mortality rates associated with COVID-19 ranging from 3.8% to 4% were reported (31, 32).

The limitations of our study are that it included a single center, that two separate RTI panels were used during the study, patients were a heterogeneous group in terms of underlying disease, the lack of vaccination information for patients and being conducted as a retrospective data analysis.

## Conclusions

A total of 313 RTI agents were identified in 185 children with hemato-oncologic disorders across 271 infectious episodes. In our study, the largest RTI outbreak occurred between November 2023 and May 2024, following the end of the COVID-19 pandemic (Figure 1; Table 2). RTI outbreaks were especially observed as a result of the reduction in non-pharmacological preventive measures. With the reopening of schools during the relaxation period, the announcement that the pandemic was over, and the lifting of the mask mandate, RTI incidence increased. This suggests that the most effective RTI control method for immunosuppressed patients remains adherence to non-pharmacological measures, such as social distancing, mask-wearing, and hand hygiene, among both patients and those in close contact with them. In our study, the most common pathogens causing RTI were *rhinovirus/enterovirus* and *influenza A/B*, and their frequencies increased significantly in the post-pandemic period. Another preventive measure against RTI is annual influenza vaccination for patients and their caregivers. In our study, the frequency of deaths related solely to RTI was low (*n* = 3). The majority of deaths occurred in patients with other comorbidities and progressive underlying disease. Clinicians should be aware that the number of viral RTI episodes increased following the pandemic related to immune debt that occurred during the lockdown period, and that agents that are not expected to be fatal may cause severe clinical conditions. It is essential to keep in mind that the most crucial step in treatment is implementing effective infection preventive measures.

## Data Availability

The raw data supporting the conclusions of this article will be made available by the authors, without undue reservation.

## References

[1] Hijano DR Maron G Hayden RT. Respiratory viral infections in patients with cancer or undergoing hematopoietic cell transplant. Front Microbiol. (2018) 9:3097. 10.3389/fmicb.2018.03097 30619176 PMC6299032

[2] Soudani N Caniza MA Assaf-Casals A Shaker R Lteif M Su Y Prevalence and characteristics of acute respiratory virus infections in pediatric cancer patients. J Med Virol. (2019) 91(7):1191–201. 10.1002/jmv.25432 30763464 PMC7166696

[3] Lefebvre M-A Rajda E Frenette C Paquet F Rubin E Sleno H Impact of the COVID-19 pandemic on healthcare-associated viral respiratory infections at a tertiary care pediatric hospital. Am J Infect Control. (2023) 51(8):961–3. 10.1016/j.ajic.2023.01.017 36736901 PMC9889274

[4] Ryoo J Kim SC Lee J. Changes in respiratory infection trends during the COVID-19 pandemic in patients with haematologic malignancy. BMC Pulm Med. (2024) 24(1):259. 10.1186/s12890-024-03071-0 38797852 PMC11129456

[5] Groves HE Piché-Renaud P-P Peci A Farrar DS Buckrell S Bancej C The impact of the COVID-19 pandemic on influenza, respiratory syncytial virus, and other seasonal respiratory virus circulation in Canada: a population-based study. Lancet Reg Health Am. (2021) 1:1–9. 10.1016/j.lana.2021.100015 PMC8285668 34386788

[6] Sarker R Roknuzzaman A Nazmunnahar Shahriar M Hossain MJ Islam MR. The WHO has declared the end of pandemic phase of COVID-19: way to come back in the normal life. Health Sci Rep. (2023) 6(9):e1544. 10.1002/hsr2.1544 37674622 PMC10478644

[7] Büyükkapu-Bay S Kebudi R Görgün Ö Meşe S Zülfikar B Badur S. Respiratory viral infections frequency and clinical outcome in symptomatic children with cancer: a single center experience from a middle-income country. Turk J Pediatr. (2018) 60(6):653–9. 10.24953/turkjped.2018.06.005 31365201

[8] Köker SA Demirağ B Tahta N Bayram N Oymak Y Karapinar TH A 3-year retrospective study of the epidemiology of acute respiratory viral infections in pediatric patients with cancer undergoing chemotherapy. J Pediatr Hematol Oncol. (2019) 41(4):e242–6. 10.1097/MPH.0000000000001418 30688827

[9] Agrawal AK Feusner J. Supportive care of patients with cancer. In: Fish JD Lipton JM Lanzkowsky P, editors. Lanzkowsky's Manual of Pediatric Hematology and Oncology (Seventh Edition). Academic Press (2022). p. 675–711. 10.1016/B978-0-12-821671-2.00020-9

[10] Demir Yenigurbuz F Atay D Akinci B Akcay A Ozturk G. Respiratory viral infections in the pediatric hematopoietic stem cell transplant population. J Pediatr Hematol Oncol. (2023) 45(1):e75–81. 10.1097/MPH.0000000000002525 36031189

[11] Agca H Akalin H Saglik I Hacimustafaoglu M Celebi S Ener B. Changing epidemiology of influenza and other respiratory viruses in the first year of COVID-19 pandemic. J Infect Public Health. (2021) 14(9):1186–90. 10.1016/j.jiph.2021.08.004 34399190

[12] Węcławek-Tompol J Zakrzewska Z Gryniewicz-Kwiatkowska O Pierlejewski F Bień E Zaucha-Prażmo A COVID-19 in pediatric cancer patients is associated with treatment interruptions but not with short-term mortality: a Polish national study. J Hematol Oncol. (2021) 14(1):163. 10.1186/s13045-021-01181-4 34635137 PMC8503711

[13] Kaçar D Kebudi R Özyörük D Tuğcu D Bahadır A Özdemir ZC Common viral respiratory infections in children with cancer during the COVID-19 pandemic: a multicenter study from Türkiye. Turk J Pediatr. (2024) 66(4):401–11. 10.24953/turkjpediatr.2024.4536 39387429

[14] Bhayana S Kalra M Sachdeva A. COVID-19 in pediatric hematology-oncology and stem cell transplant patients–the spectrum of illness, complications and comparison of first two waves. Pediatr Hematol Oncol J. (2022) 7(3):96–102. 10.1016/j.phoj.2022.05.003

[15] Kuitunen I Artama M Mäkelä L Backman K Heiskanen-Kosma T Renko M. Effect of social distancing due to the COVID-19 pandemic on the incidence of viral respiratory tract infections in children in Finland during early 2020. Pediatr Infect Dis J. (2020) 39(12):e423–7. 10.1097/INF.0000000000002845 32773660

[16] Li Y Wu Z Yan Y Shi Y Huang J Du H Prevalence of respiratory viruses among hospitalized children with lower respiratory tract infections during the COVID-19 pandemic in Wuhan, China. Int J Infect Dis. (2024) 139:6–12. 10.1016/j.ijid.2023.11.019 37984762

[17] Vahlkvist S Mohammad A Kofoed PE. The impact of viral co-infection in children treated with respiratory support due to lower respiratory tract infections. An observational study. Pediatr Pulmonol. (2025) 60(1):e27467. 10.1002/ppul.27467 39760453

[18] Khales P Razizadeh MH Ghorbani S Moattari A Saadati H Tavakoli A. Prevalence of respiratory viruses in children with respiratory tract infections during the COVID-19 pandemic era: a systematic review and meta-analysis. BMC Pulm Med. (2025) 25(1):135. 10.1186/s12890-025-03587-z 40133851 PMC11934662

[19] Buonsenso D Ferro V Viozzi F Morello R Proli F Bersani G Changes in clinical, demographic, and outcome patterns of children hospitalized with non-SARS-CoV-2 viral low respiratory tract infections before and during the COVID pandemic in Rome, Italy. Pediatr Pulmonol. (2024) 59(2):362–70. 10.1002/ppul.26755 37937896

[20] Lee H Lee H Song K-H Kim ES Park JS Jung J Impact of public health interventions on seasonal influenza activity during the COVID-19 outbreak in Korea. Clin Infect Dis. (2021) 73(1):e132–40. 10.1093/cid/ciaa672 32472687 PMC7314207

[21] Zhao X Zhu X Wang J Ye C Zhao S. The epidemiological analysis of respiratory virus infections in children in Hangzhou from 2019 to 2023. Virus Res. (2025) 355:199558. 10.1016/j.virusres.2025.199558 40088949 PMC11979517

[22] Barr RS Drysdale SB. Viral respiratory tract infections in the immunocompromised child. Pediatr Infect Dis J. (2023) 42(5):e170–2. 10.1097/INF.0000000000003855 36795556 PMC10097468

[23] Zhao P Zhang Y Wang J Li Y Wang Y Gao Y Epidemiology of respiratory pathogens in patients with acute respiratory infections during the COVID-19 pandemic and after easing of COVID-19 restrictions. Microbiol Spectr. (2024) 12(11):e01161–24. 10.1128/spectrum.01161-24 39320069 PMC11537120

[24] Kebudi R Kurucu N Tuğcu D Hacısalihoğlu Ş Fışgın T Ocak S COVID-19 infection in children with cancer and stem cell transplant recipients in Turkey: a nationwide study. Pediatr Blood Cancer. (2021) 68(6):e28915. 10.1002/pbc.28915 33538100 PMC7995085

[25] Bisogno G Provenzi M Zama D Tondo A Meazza C Colombini A Clinical characteristics and outcome of severe acute respiratory syndrome coronavirus 2 infection in Italian pediatric oncology patients: a study from the infectious diseases working group of the associazione italiana di oncologia e ematologia pediatrica. J Pediatric Infect Dis Soc. (2020) 9(5):530–4. 10.1093/jpids/piaa088 32652521 PMC7454778

[26] Boulad F Kamboj M Bouvier N Mauguen A Kung AL. COVID-19 in children with cancer in New York city. JAMA Oncol. (2020) 6(9):1459–60. 10.1001/jamaoncol.2020.2028 32401276 PMC7221844

[27] Millen GC Arnold R Cazier J-B Curley H Feltbower R Gamble A COVID-19 in children with haematological malignancies. Arch Dis Child. (2022) 107(2):186–8. 10.1136/archdischild-2021-322062 34301621 PMC8785070

[28] Chiu N-C Chi H Lin C-Y. Targeting high-risk groups in the post-pandemic era: a focus on pediatric hematological malignancies. Pediatr Neonatol. (2024) 65(6):525–6. 10.1016/j.pedneo.2024.08.003 39242226

[29] Ebeid FSE El-Din AMK Magdi SM Abd El Kader HM Aly NH. Clinico-Epidmological study and outcome of COVID 19 infection in children with hemato-oncological disorders. QJM. (2024) 117(Supplement_2):hcae175.777. 10.1093/qjmed/hcae175.777

[30] Wang W Xu X Bai S Wang L Luo J Zhao D Clinical characteristics and prognosis of SARS-CoV-2 infection in children with hematological malignancies: a multicenter, retrospective study in China. Pediatr Neonatol. (2024) 65(6):553–9. 10.1016/j.pedneo.2023.12.006 38553357

[31] Vijenthira A Gong IY Fox TA Booth S Cook G Fattizzo B Outcomes of patients with hematologic malignancies and COVID-19: a systematic review and meta-analysis of 3377 patients. Blood J Am Soc Hematol. (2020) 136(25):2881–92. 10.1182/blood.2020008824 PMC7746126 33113551

[32] Mukkada S Bhakta N Chantada GL Chen Y Vedaraju Y Faughnan L Global characteristics and outcomes of SARS-CoV-2 infection in children and adolescents with cancer (GRCCC): a cohort study. Lancet Oncol. (2021) 22(10):1416–26. 10.1016/S1470-2045(21)00454-X 34454651 PMC8389979

